# New Public Health and Sport Medicine Institutions Guidelines of Physical Activity Intensity for Pregnancy—A Scoping Review

**DOI:** 10.3390/jcm13061738

**Published:** 2024-03-18

**Authors:** Aneta Worska, Ida Laudańska-Krzemińska, Julia Ciążyńska, Beata Jóźwiak, Janusz Maciaszek

**Affiliations:** Department of Physical Activity and Health Promotion Science, Poznan University of Physical Education, 61-871 Poznan, Poland; idakrzeminska@awf.poznan.pl (I.L.-K.); ciazynska@awf.poznan.pl (J.C.); b.jozwiak@awf.poznan.pl (B.J.); jmaciaszek@awf.poznan.pl (J.M.)

**Keywords:** guidelines, recommendations, intensity, physical activity, exercise, pregnancy

## Abstract

**Background:** Before starting PA, pregnant women should select the appropriate type of training and adjust its components to the development of pregnancy and her capabilities. This review aimed to analyze current recommendations for pregnant women on methods for determining and assessing PA intensity levels and characterize the extent and nature of the information provided to pregnant women in official documents published by public health and sports medicine institutions. **Methods:** The review was conducted as per the PRISMA Extension for Scoping Reviews (PRISMA-ScR). We searched scientific databases (PubMed, ScienceDirect, Web of Science, Academic Search Complete, and SPORTDiscus with Full Text via EBSCO) and the Internet to identify papers regarding recommendations for the PA intensity level for pregnant women. We analyzed 22 eligible guidelines, published over the last 10 years in English, from nine countries and three international organizations. **Results:** The PA of pregnant women should be at a moderate level. As for higher levels, the analyzed recommendations are contradictory. Methods for assessing PA intensity levels are often not included. The most frequently recommended methods for determining and assessing the PA intensity level for pregnant women are the rating of perceived exertion, the Talk Test, and heart rate measurements. Few guidelines offer specific advice for highly active women (e.g., elite athletes) or trimester-specific considerations. **Conclusions:** The number of published recommendations regarding PA during pregnancy has increased over the last decade. The amount of information on PA intensity levels is still insufficient. There is a need to update them, based on high-quality scientific work.

## 1. Introduction

With a proper course of pregnancy and proper dosing, physical activity (PA) has a positive effect on the mother and child’s body. Regular moderate-intensity PA helps to maintain a good psychophysical condition and prevents excessive weight gain during pregnancy. Physically active pregnant women tend to have lower anxiety and stress levels. In addition, they prepare their body for the effort associated with childbirth and subsequent care of the newborn. The period of regeneration of the body during the postpartum period is faster for them [1,2,3,4,5,6].

PA is defined as any body movement produced by skeletal muscles that results in energy expenditure [7,8]. It is often used interchangeably with the word “exercise”, although they are not the same thing. Exercise is a planned, repetitive, and organized element of PA. Although PA recommendations should always be adapted to the individual needs and capabilities of the exerciser, there are common elements of every training: frequency, duration, time, type, and intensity. This paper focuses on intensity and methods for determining its level.

Changes in physiological and metabolic profiles depend on the PA intensity level. The intensity is determined by goal, age, abilities, preferences, and level of physical fitness. A certain level of effort is necessary, but it cannot exceed the endurance threshold of the cardiorespiratory and musculoskeletal systems [9]. Various methods can be used to determine the PA intensity level, but the most frequently used ones concern heart rate reserve (%HRR), oxygen uptake reserve (VO_2_R) [10], or metabolic equivalents (METs) [11].

The increased metabolism associated with pregnancy (both resting and during exercise) puts a strain on the respiratory and cardiovascular systems due to the increased oxygen demand. Cardiac output (Q) increases 30% to 50%. The pregnancy-related increase in Q in the first trimester is mainly due to a 10% increase in stroke volume, while the increase in heart rate (HR) in the second and third trimesters is mainly responsible for maintaining Q at its maximum level [11].

Given the variability of a pregnant woman’s HR, other valid, self-reported but much simpler and more practical methods of assessing PA intensity levels can be used [10,12], such as scales used for the rating of perceived exertion (RPE) or the Talk Test. RPE values correlate closely with objectively measured symptoms of fatigue measured by HR values. Special attention should be paid to ensure that the exerciser reports the total amount of load and fatigue experienced and not individual symptoms accompanying the exercise (muscle pain, breathing difficulties, etc.) [13]. The first exemplary method based on a subjective assessment of fatigue levels created by Swedish physiologist Gunner Borg is the Borg RPE scale. For moderate PA, ratings should be 13 to 14 (somewhat hard) on the Borg RPE scale (Table 1), where 6 represents no exertion and 20 represents maximal exertion [13].

Another method that can be used to monitor the subjective feeling of the PA intensity level is the Talk Test, which was created based on the advice given by Professor John Grayson at Oxford University in 1939 to British mountaineers to “climb no faster than you can speak”. It assumes that most health benefits are associated with moderate-intensity PA, which is described as the one that allows the exerciser to talk freely [14]. Vigorous PA is associated with substantial increases in breathing and the inability to carry on a normal conversation easily [15].

A study conducted in 2014 analyzed seventeen guidelines on physical activity for pregnant women [16]. It was found that only nine of these included information on methods for determining the intensity of physical activity. The most frequently recommended methods reported were the Borg RPE scale, the Talk Test, and heart rate measurement. The study highlighted the necessity for a wider dissemination of these tools among both pregnant women and healthcare professionals [16].

Some healthcare providers have reported concerns about the advice they give their patients about PA [17,18]. This is not surprising as pregnant women characterize healthcare providers’ advice about PA as overly conservative, vague, and confusing [19,20,21]. Every pregnancy is different, so healthcare providers, pregnant women or exercise specialists need to know and be able to determine the appropriate PA intensity, individually, for a pregnant woman’s abilities, preferences, and needs.

The term “method for determining and assessing PA intensity” includes all methods and tools for determining the recommended range of PA intensity and monitoring it during PA. The main purpose of this review is to analyze current recommendations for pregnant women on methods for determining and assessing PA intensity levels. Our goal is also to see if anything has changed, and thus improved, over the past decade in determining PA intensity levels in pregnant women. Our goal is not to test the accuracy of the recommendations or their effectiveness. Rather, our study seeks to characterize the extent and nature of information provided to pregnant women in official documents published by public health and sports medicine institutions. Our goal is to identify areas that can be improved or enhanced and to highlight opportunities for future research.

## 2. Materials and Methods

### 2.1. Identifying the Research Question

In our study, we identified four questions:

1. What PA intensity level is currently recommended for pregnant women?
2. What methods of monitoring the PA intensity level are currently recommended for pregnant women?
3. What has improved over the past decade in determining the PA intensity level for pregnant women?
4. Do the recommendations provide specific information on the PA intensity level for pregnant women?

### 2.2. Criteria for Including Studies

The review was conducted as per the PRISMA Extension for Scoping Reviews (PRISMA-ScR) [22]. Studies from 2014 to November 2023 were considered for inclusion based on inclusion/exclusion criteria (Table 2).

**Figure 1:**
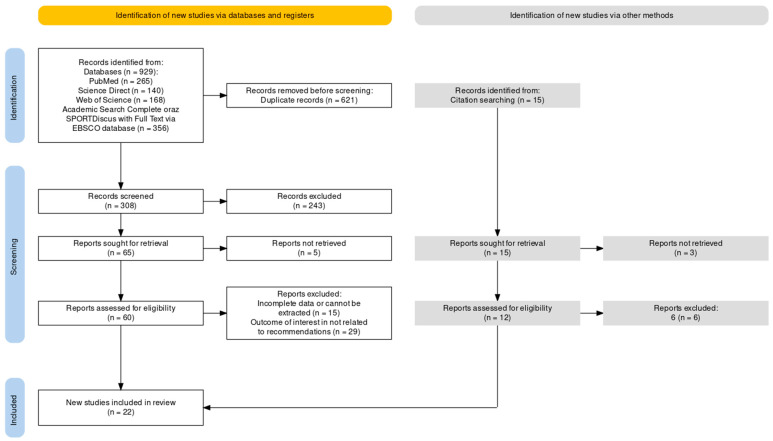
Overview of the screening and paper selection process (PRISMA flowchart) [23].

Figure 1 shows the PRISMA diagram of the article screening process.

### 2.3. Information Sources

In the first stage of searching for materials for analysis, PubMed, ScienceDirect, Web of Science, Academic Search Complete, and SPORTDiscus with Full Text via EBSCO databases were used. Whereas the guidelines do not have to be of the nature of scientific work but only constitute the official position of institutions representative of a given country, in the second stage, the publicly available Internet was searched. Key terms used in searching electronic databases were “pregnancy”, “physical activity”, or “exercise”, “recommendations”, or “guidelines”. The search was carried out in November 2023.

### 2.4. Selection of Studies

After collating the search results, we filtered out any duplicates to ensure a unique set of studies. In the next step, two evaluators rigorously assessed the titles of the studies against specific inclusion and exclusion parameters listed in Table 2. Titles that were indicative of potential relevance to our research criteria led to a thorough inspection of the study abstracts. Studies with abstracts that indicated possible compliance with the inclusion criteria had their full texts retrieved for detailed examination. To be comprehensive, we cross-referenced the citations within these papers to identify any pertinent studies that might have been overlooked in our original search. Finally, with meticulous attention to detail, two researchers (AW and JC) independently analyzed these full texts, once again employing the inclusion and exclusion criteria.

### 2.5. Data Charting

The data from selected articles were extracted in a Microsoft Excel 365 sheet independently by two investigators into a form designed after deliberations by all the authors. The following data were charted: authors or publishing organizations, country, year of publication, title, recommended level of PA intensity in pregnancy (divided into two subsections: general recommendations and detailed recommendations), safe upper limit of PA intensity, PA intensity determining and monitoring methods (divided into five subsections: VO_2max_, HR range, MET, the Borg RPE scale/RPE scale, the Talk Test), PA intensity depending on PA experience (divided into three subsections: inactive before pregnancy, regular physical activity before pregnancy, athletes), and additional information on the PA intensity (divided into three subsections: PA intensity depending on the trimester of pregnancy, PA intensity depending on altitudes, and other). Table 3, as an abbreviated version of Supplementary S1, shows all documents included in this review, including authors or publishing organizations, country, year of publication, recommended PA intensity—general recommendations, recommended PA intensity—detailed recommendations, whether information about the safe upper limit of PA intensity is included, PA intensity determining and monitoring methods, whether information about PA intensity depending on PA experience is included, and whether other information related to the PA intensity is included.

## 3. Results

### 3.1. Study Characteristics

A systematic search of public health and sports medicine institutions’ guidelines for pregnancy identified a total of 929 papers for evaluation. After further exclusion (the rejection criteria are included in Table 1) and removing duplicates and papers irrelevant to the selected topic, 16 papers were selected for data charting [24,25,26,27,28,29,32,33,38,39,40,41,42,43,44,45]. Based on the reference lists presented in these papers, an additional six studies were included [30,31,34,35,36,37]. Four studies were conducted in Australia, one each in Australia and New Zealand, Austria, Canada, Cyprus, Poland, and Portugal, three in the United Kingdom, four in the United States, and five by international organizations.

### 3.2. Outcomes

Twenty-two documents containing recommendations on exercise during pregnancy from nine countries were analyzed: Australia, Australia and New Zealand, Austria, Canada, Cyprus, Poland, Portugal, the United Kingdom, the United States, and three international organizations: European Board and College of Obstetrics and Gynecology (EBCOG), International Olympic Committee (IOC) and World Health Organization (WHO), looking for information on the recommended PA intensity level and methods of determining and assessing it during classes (Table 2).

**Figure 2:**
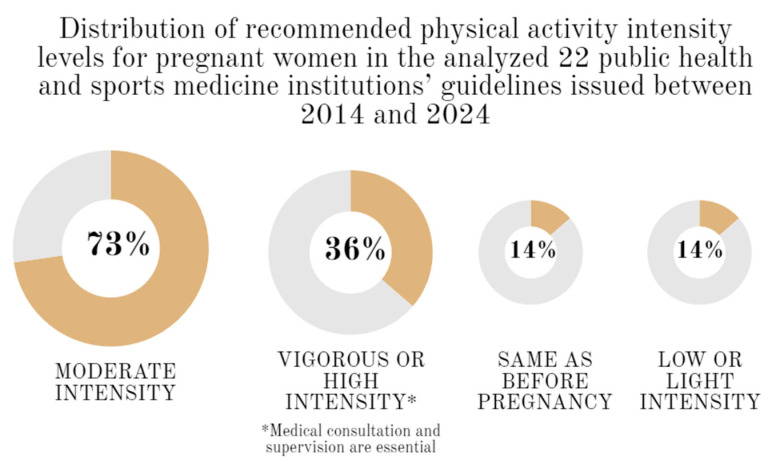
Distribution of recommended physical activity intensity levels for pregnant women in the analyzed 22 public health and sports medicine institutions’ guidelines issued between 2014 and 2024.

All recommendations apply to healthy women during an uncomplicated pregnancy, regardless of whether they were regularly physically active before pregnancy or physically inactive. Only one of them contained additional information about obese women [33]. Among the twenty-two guidelines reviewed for physical activity in pregnant women, the one issued by Portugal lacked any details regarding the intensity of the physical activity [36]. According to the remaining twenty-one, a pregnant woman should exercise at moderate intensity [24,25,26,27,28,29,30,31,32,33,34,35,37,38,39,40,41,42,43,44,45]. Ten recommendations included information on vigorous or high intensity [26,27,29,30,33,37,39,41,43,44]. Three guidelines recommend PA intensity at the same level as before pregnancy [31,40,45]. Figure 2 illustrates the percentage distribution of recommendations by three levels of intensity with an additional category of “same as before pregnancy.” Elite athletes or active women can take up PA with higher intensity under strict medical supervision [24,28,29,33,37,40]. Only one document specified that elite athletes are recommended to refrain from training at intensities higher than 90% of their VO_2max_ [28]. Moreover, only one recommendation claimed that vigorous-intensity exercise completed into the third trimester appears to be safe for most athletes with healthy pregnancies [33].

The analyzed documents also included information that high-intensity training at altitudes of more than 1500–2000 m above sea level is not recommended [25,27,28].

Additionally, vigorous activity is not recommended for previously inactive women [35]; one recommendation explicitly advised against high-intensity exercise sports [30], and one recommended caution [41]. A similar provision was found in the American [24] recommendations, which advised not to increase the intensity of PA during pregnancy compared with the pre-conception period [40]. In 2016, the International Olympic Committee concluded that high-intensity training can be harmful to the fetus [26].

In contrast, three guidelines [43,44,45] suggested an alternative approach. They indicated that women who have a background in regular exercise may continue with high-intensity physical activities if the pregnancy is progressing healthily and they undergo consistent monitoring [43]. For those women with a higher level of fitness who are accustomed to regular vigorous exercises, a rating of 15–16 of the Borg PRE scale (equating to “hard”) may be appropriate [44], and in the third trimester, no more than three sessions per week of vigorous exercise are recommended [45].

**Figure 3:**
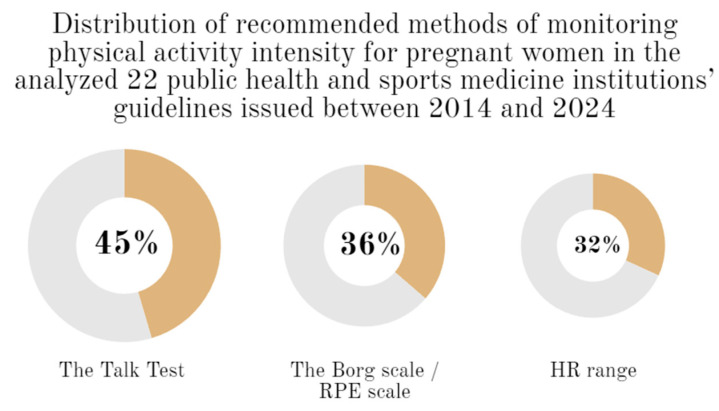
Distribution of recommended methods of monitoring physical activity intensity for pregnant women in the analyzed 22 public health and sports medicine institutions’ guidelines issued between 2014 and 2024.

The most frequently mentioned methods for determining and assessing intensity levels were: the Borg RPE scale (or RPE scale) [25,27,28,33,37,43,44,45], the Talk Test [27,29,32,33,37,38,39,42,43,44], and the use of tables with exercise HR ranges for pregnant women [29,33,35,38,43,44,45]. Figure 3 illustrates the percentage distribution of recommendations by the methods cited in the documents for monitoring physical activity intensity.

The exercise HR range depends on the woman’s age, training level, or body mass index (BMI) and was mentioned in seven papers [29,33,35,38,43,44,45]. According to Canadian guidelines, the PA intensity during pregnancy should remain at the level of 40% to 59% HRR and a vigorous-intensity PA level of 60% to 80% HRR [29]. They recommend relying on the modified HR ranges for aerobic exercise during pregnancy which are dependent on the age of the pregnant woman (maximal maternal HR). For women aged 29 years old and younger, the range of HR beats per minute for low PA intensity is 102–124, for moderate PA intensity, it is 125–146, and for vigorous PA intensity, it is 147–169. For women aged 30 years old and older, the range of HR beats per minute for low PA intensity is 101–120, for moderate PA intensity, it is 121–141, and for vigorous PA intensity, it is 142–162 [29]. The analyzed documents recommend relying on the modified HR ranges for aerobic exercise during pregnancy [29] or to keep below 60–80% of the age-predicted maximum maternal HR [33]. British recommendations advise women who exercised more intensively before pregnancy to work at 50–70% of their maximum maternal HR [35]. A more conservative approach is presented by the WHO, which recommends the use of HR as a determinant of moderate intensity but defines it as “raise your heart rate, and make you breathe faster” without giving specific HR ranges [38]. Only two of the analyzed guidelines included information that there were no clear-cut data on the impact of maximum PA at a level above 90% of the maximum HR on pregnancy [33,43]. The guidelines from Canada, Britain, and Poland propose that women accustomed to high levels of training can employ heart rate monitoring as a tool to gauge the intensity of their physical activities [29,35,43].

The guidelines of seven documents recommend that PA intensity during pregnancy should be moderate [24,26,30,31,34,38,41]. However, they lack information on how a given intensity can be determined and monitored.

No document proposed determining PA intensity based on the maximum minute oxygen uptake (VO_2max_). Also, in none of them, PA intensity was defined in metabolic equivalents (METs).

The American and Polish guidelines admit that a safe upper limit of PA intensity for pregnant women has not been established [33,43].

## 4. Discussion

### 4.1. About the Findings

The analyzed documents were issued after 2014. Since then, there have been many scientific papers on the subject of prenatal physical activity, including exercise intensity, which should be the basis for updating the guidelines in different countries. Nevertheless, these documents have had a positive impact on the perception of physical activity among pregnant women over the last 10 years. The differences in the recommendations in the analyzed guidelines showed how controversial the issue of PA intensity during pregnancy is. Although no upper limit for PA intensity during pregnancy has been established so far [33], women tend to reduce it.

The restriction of maternal HR during PA in pregnancy was first introduced with the inaugural American College of Obstetricians and Gynecologists (ACOG) guidance issued in 1985 [46]. The guidance indicated that exercise target HR for pregnant women should be set 25–30% lower than at other times and should not exceed 140 beats per minute. It is not clear how the value of 140 beats per minute was originally obtained [46]. The HR recommendation was removed in the next update from ACOG in 1994 [47] and in 2002 [48]. Despite the removal of HR recommendations, healthcare providers continued advising based on the 1985 ACOG guidelines using the HR restriction of 140 bpm [17,18]. According to American guidelines, because blunted and normal HR responses to PA have been reported in pregnant women, the use of the RPE scale may be a more effective means to monitor PA intensity levels during pregnancy than HR parameters [33].

Another example of contradictions in the recommendations contained in the PA guidelines for pregnant women is the fact that ACOG encourages healthy pregnant women to perform regular, moderate exercise without defining what should be understood as “moderate exercise” [33]. A definition of moderate intensity was found in only 7 of the 22 documents analyzed [24,25,29,32,35,38,40]. In non-pregnant populations, Health and Human Services defines “moderate” exercise as: “(1) activity requiring 3–5.9 metabolic equivalents (METs); (2) 40–59% of aerobic capacity or heart rate reserve (i.e., target heart rate, THR); or (3) rating of perceived exertion (RPE) of 5–6 on 10-point scale” [49]. Szymanski and Satin’s study evaluates the use of METs, age-predicted THRs, and RPE compared to individualized THRs derived from an exercise test for prescribing moderate exercise. They concluded that despite accurate THR values, this may not be practical in the context of moderate-intensity PA, because it may cause “women to work harder than necessary”. Also, the RPE scale was not an accurate method. When women exercised at their individualized THR values, their average MET levels were in the moderate range. Therefore, the researchers conclude that MET may be a practical method of instructing pregnant women on “moderate” PA.

The International Olympic Committee (IOC) stated that high-intensity training can be harmful to the fetus [26]. A different position is presented by the Canadian [29], American [33], Australian [37], and Polish [43] recommendations, as well as by one international institution, namely EBCOG [29,33,37,41]. According to their recommendations, women can take up PA with higher intensity, under strict medical supervision or with caution. Based on a review of the scientific literature, performing well-structured high-intensity training during pregnancy is safe in terms of obstetric outcomes and well tolerated by pregnant women [50]. High-intensity training, exemplified by the high-intensity interval training (HIIT) intervention, either led to improvements in selected maternal and fetal health parameters or had no effect [51]. No adverse effects were observed. Moreover, researchers confirm not only the safety of HIIT but also many health effects on the physiological, biochemical, and psychological parameters of future mothers [52,53].

According to the analyzed guidelines, all women with normal pregnancies can continue their PA using pre-pregnancy weights or start a new training program [31,40,43,45]. Polish recommendations indicate that the intensity and type of exercise should be individualized [43]. Based on the recommendations analyzed above, it is difficult to indicate how intense a pregnant woman’s physical activity should be and, most importantly, how to measure it.

### 4.2. Limitations

We have attempted to take a systematic and iterative approach to identifying and synthesizing guidelines aimed not only at pregnant women and the perinatal health care professionals who support them but also to ensure that these guidelines are available to the broader public domain. Even though efforts were made to incorporate maximum papers in the review, some relevant documents may have been overlooked. For example, documents reported in languages other than English were not included. Moreover, documents issued by institutions other than government, medical, or sports institutions were excluded.

Because available recommendations regarding exercise during pregnancy not always have the character of scientific work and more often constitute an official representative position of an institution, it was, in some cases, impossible to find them in scientific databases. Nevertheless, the authors tried to adopt such a procedure when searching for material for analysis that would make it effective and repeatable while being aware that some documents that met the criteria for inclusion in the analysis could have been omitted.

## 5. Conclusions

1. Over the last 10 years, many new guidelines for PA during pregnancy have appeared. Some of them contain contradictory recommendations.
2. A moderate intensity level of physical activity is most commonly recommended for pregnant women and sometimes vigorous intensity also. Recommendations for a high intensity level of physical activity are contradictory in different countries. They need to be updated on the basis of high-quality scientific and research studies.
3. The guidelines lack information on individualized methods of determining the intensity of PA during pregnancy, preceded by an assessment of the physical performance of pregnant women. They do not provide methods for determining the maximal oxygen uptake, either by indirect or direct measurements. This needs to be updated.
4. The most frequently recommended methods of determining and assessing the intensity of PA in pregnant women are the rating of perceived exertion scale—RPE, the Talk Test, and reference to tables with HR exercise ranges for pregnant women. These tools should be disseminated among pregnant women, prenatal physical activity specialists, and obstetricians and midwives.

## Figures and Tables

**Table 1:** Borg rating of perceived exertion (RPE) 6–20 scale [13].

Rating	Perceived Exertion
20	Maximal exertion
19	Extremely hard
17–18	Very hard
15–16	Hard
13–14	Somewhat hard
10–12	Light
8–9	Very light
7	Extremely light
1–6	No exertion at all

**Table 2:** Inclusion/exclusion criteria for the selection of papers.

Inclusion	Exclusion
Guidelines must have been published in English	Other language papers were excluded
Guidelines must have been published after 2014	Reviews, editorials, commentaries, and qualitative studies were excluded
Guidelines published by either public health or sports medicine institutions	Documents without access to all content were excluded

**Table 3:** Short characteristics of analyzed guidelines on physical activity intensity in pregnancy and methods of determining and assessing it during classes, published after 2014.

Authors or Publishing Organizations	Country	Year of Publication	Recommended PA Intensity—General Recommendations ^1^	Recommended PA Intensity—Detailed Recommendations ^2^	The Safe Upper Limit of PA Intensity	PA Intensity Determining and Monitoring Methods	PA Intensity Depending on PA Experience	Additional Information on the PA Intensity
American College of Nurse-Midwives (ACNM) [24]	United States	2014	moderate	not included	not included	not included	included	included
International Olympic Committee (IOC) [25]	international	2016	not included	not included	not included	the Borg scale/RPE scale	included	included
International Olympic Committee (IOC) [26]	international	2016	light to moderate; high or can be harmful	not included	not included	not included	not included	not included
Sport Medicine Australia (SMA) [27]	Australia	2016	moderate to vigorous	included (aerobic activities and muscle-strengthening exercises)	not included	the Borg scale/RPE scale; the Talk Test	included	included
International Olympic Committee (IOC) [28]	international	2018	light to moderate	not included	not included	the Borg scale/RPE scale	included	included
Society of Obstetricians and Gynecologists of Canada; Canadian Society for Exercise Physiology SOGC/CSEP [29]	Canada	2018	moderate; higher under strict medical supervision	not included	not included	HR range; the Talk Test	included	not included
Cyprus National Addictions Authority [30]	Cyprus	2019	low to moderate	include (cardio exercises)	not included	not included	included	not included
Department of Health and Social Care, Scottish Government [31]	United Kingdom	2019	the same as before pregnancy	not included	not included	not included	included	not included
Exercise is Medicine/American College of Sports Medicine (EIM/ACSM) [32]	United States	2019	moderate	not included	not included	the Talk Test	not included	not included
American College of Obstetricians and Gynecologists (ACOG) [33]	United States	2020	moderate; higher under strict medical supervision; obesity: low to comfortable	not included	is unknown	HR range; the Borg scale/RPE scale; the Talk Test	included	included
Austrian Health Promotion Fund [34]	Austria	2020	moderate	not included	not included	not included	not included	not included
British Association of Sport and Exercise Medicine [35]	United Kingdom	2020	moderate	not included	not included	HR range	included	included
Casa do Brasil de Lisboa [36]	Portugal	2020	not included	not included	not included	not included	not included	not included
Department of Health Australian Government [37]	Australia	2020	moderate to vigorous; higher after consulting a doctor	not included	not included	the Borg scale/RPE scale the Talk Test	included	not included
World Health Organization (WHO) [38]	international	2020	moderate	not included	not included	HR range; the Talk Test	included	not included
Australian Government. Department of Health (AGDH) [39]	Australia	2021	moderate to vigorous	not included	not included	the Talk Test	not included	not included
American College of Sports Medicine (ACSM) [40]	United States	not reported, access in 2023	the same as before pregnancy	not included	not included	not included	included	not included
European Board and College of Obstetrics and Gynecology (EBCOG) [41]	International	2023	moderate; higher with caution	not included	not included	not included	not included	included
National Health Service NHS [42]	United Kingdom	2023	not included	not included	not included	the Talk Test	not included	not included
Polish Society of Gynecologists and Obstetricians (PTGiP) and Polish Society of Sports Medicine (PTMS) [43]	Poland	2023	moderate to high	included (muscle strengthening, balance, neuromotor, and pelvic floor exercises)	is unknown	HR range; the Borg scale/RPE scale; the Talk Test	included	included
Royal Australian and New Zealand College of Obstetricians and Gynecologists (RANZCOG) [44]	Australia and New Zealand	2023	moderate to vigorous	not included	not included	HR range; the Borg scale/RPE scale; the Talk Test	included	included
Sport Medicine Australia (SMA) [45]	Australia	not reported, access in 2023	the same as before pregnancy	not included	not included	HR range; the Borg scale/RPE scale	not included	not included

^1^ Information on the recommended level of PA intensity. ^2^ Information on PA intensity related to the particular elements of the PA for pregnant women (flexibility, muscular strength, and cardio-vascular fitness elements).

## Data Availability

Not applicable.

## Supplementary Materials

The following supporting information can be downloaded at: https://www.mdpi.com/article/10.3390/jcm13061738/s1, Supplementary S1: Overview table with details of all documents selected for this review, including details about the documents, study objectives, and study outcomes.
